# Retraction: Impact of Microplastic Exposure on Human Health: A Systematic Review of Mechanisms, Biomarkers, and Clinical Outcomes

**DOI:** 10.7759/cureus.r251

**Published:** 2026-10-08

**Authors:** Avrina K Ririe, Nusrat Fatema, Tanni J Dina, Jyothsna Devi Kuchipudi, Prima Tamanna, Libriansyah Libriansyah, Tahmina Akter, Nafisa Kaderi

**Affiliations:** 1 Pathology and Laboratory Medicine, David Geffen School of Medicine (DGSOM), University of California Los Angeles (UCLA), Los Angeles, USA; 2 Public Health, Popular Medical College, Dhaka, BGD; 3 Public Health, Sheikh Hasina Medical College and Hospital, Tangail, BGD; 4 Area of Nutrition and Dietetics, Jain University School of Allied Healthcare and Science (Deemed-to-be-University), Bengaluru, IND; 5 Public Health, National Institute of Preventive and Social Medicine (NIPSOM), Rangpur, BGD; 6 Public Health, Prime Medical College, Rangpur, BGD; 7 Division of Endocrinology, Metabolism, and Diabetes, Rumah Sakit Pusat Angkatan Laut (RSPAL) Dr. Ramelan (Naval Hospital), Surabaya, IDN; 8 Dentistry/Preventive Medicine, City Dental College and National Institute of Preventive and Social Medicine, Dhaka, BGD; 9 Public Health, Shaheed M. Monsur Ali Medical College, Sirajganj, BGD

This article has been retracted by the Editor-in-Chief due to concerns regarding the reliability of the data extracted from the included studies. After publication, concerns were raised that the study characteristics reported for included references do not correspond to the content of those references, and the conclusions of the review therefore cannot be relied upon. The authors did not respond to the journal's correspondence.

